# Atmospheric conditions create freeways, detours and tailbacks for migrating birds

**DOI:** 10.1007/s00359-017-1181-9

**Published:** 2017-05-15

**Authors:** Judy Shamoun-Baranes, Felix Liechti, Wouter M. G. Vansteelant

**Affiliations:** 1 Theoretical and Computational Ecology, Institute for Biodiversity and Ecosystem Dynamics, University of Amsterdam, P.O. Box 94248, 1090 GE Amsterdam, The Netherlands; 2 Department of Bird Migration, Swiss Ornithological Institute, Seerose 1, 6204 Sempach, Switzerland; 3 Vansteelant Eco Research, Dijkgraaf 35, 6721 NJ Bennekom, The Netherlands

**Keywords:** Birds, Biologging, Flight behaviour, Radar, Weather

## Abstract

The extraordinary adaptations of birds to contend with atmospheric conditions during their migratory flights have captivated ecologists for decades. During the 21st century technological advances have sparked a revival of research into the influence of weather on migrating birds. Using biologging technology, flight behaviour is measured across entire flyways, weather radar networks quantify large-scale migratory fluxes, citizen scientists gather observations of migrant birds and mechanistic models are used to simulate migration in dynamic aerial environments. In this review, we first introduce the most relevant microscale, mesoscale and synoptic scale atmospheric phenomena from the point of view of a migrating bird. We then provide an overview of the individual responses of migrant birds (when, where and how to fly) in relation to these phenomena. We explore the cumulative impact of individual responses to weather during migration, and the consequences thereof for populations and migratory systems. In general, individual birds seem to have a much more flexible response to weather than previously thought, but we also note similarities in migratory behaviour across taxa. We propose various avenues for future research through which we expect to derive more fundamental insights into the influence of weather on the evolution of migratory behaviour and the life-history, population dynamics and species distributions of migrant birds.

## Introduction

**Fig. 1:**
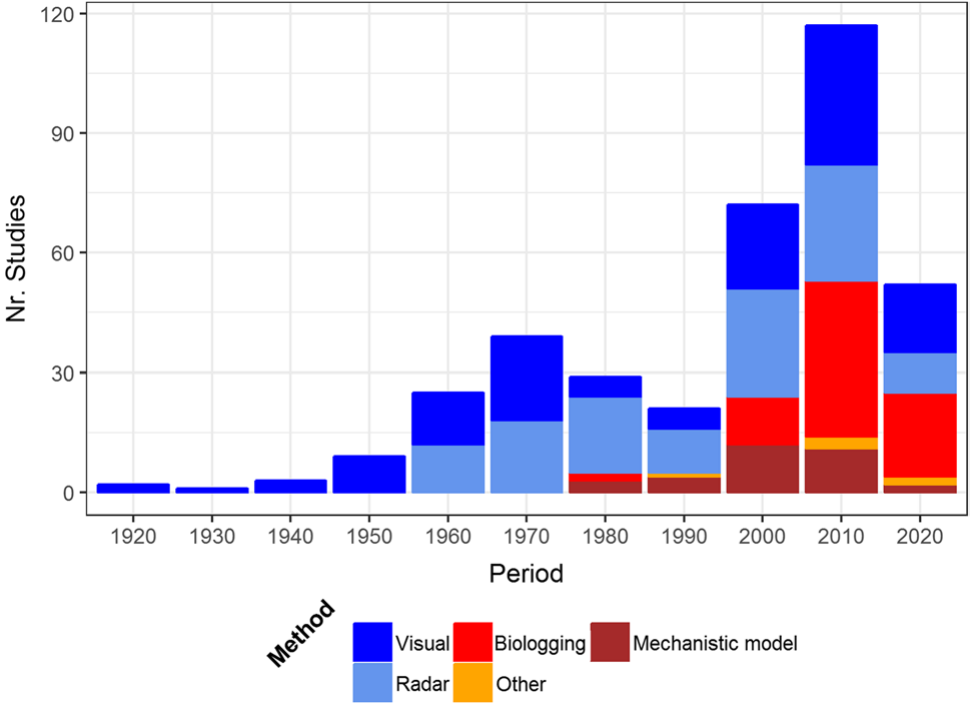
Temporal overview of studies on the influence of weather on bird migration over time. Studies are broken down into the main methods that were used, see “Methods” for a detailed description of how different methods were classified. The number of studies was binned by rounding the year to the nearest decade, i.e. 1920 represents all studies from 1915 to 1924 and so on

**Fig. 2:**
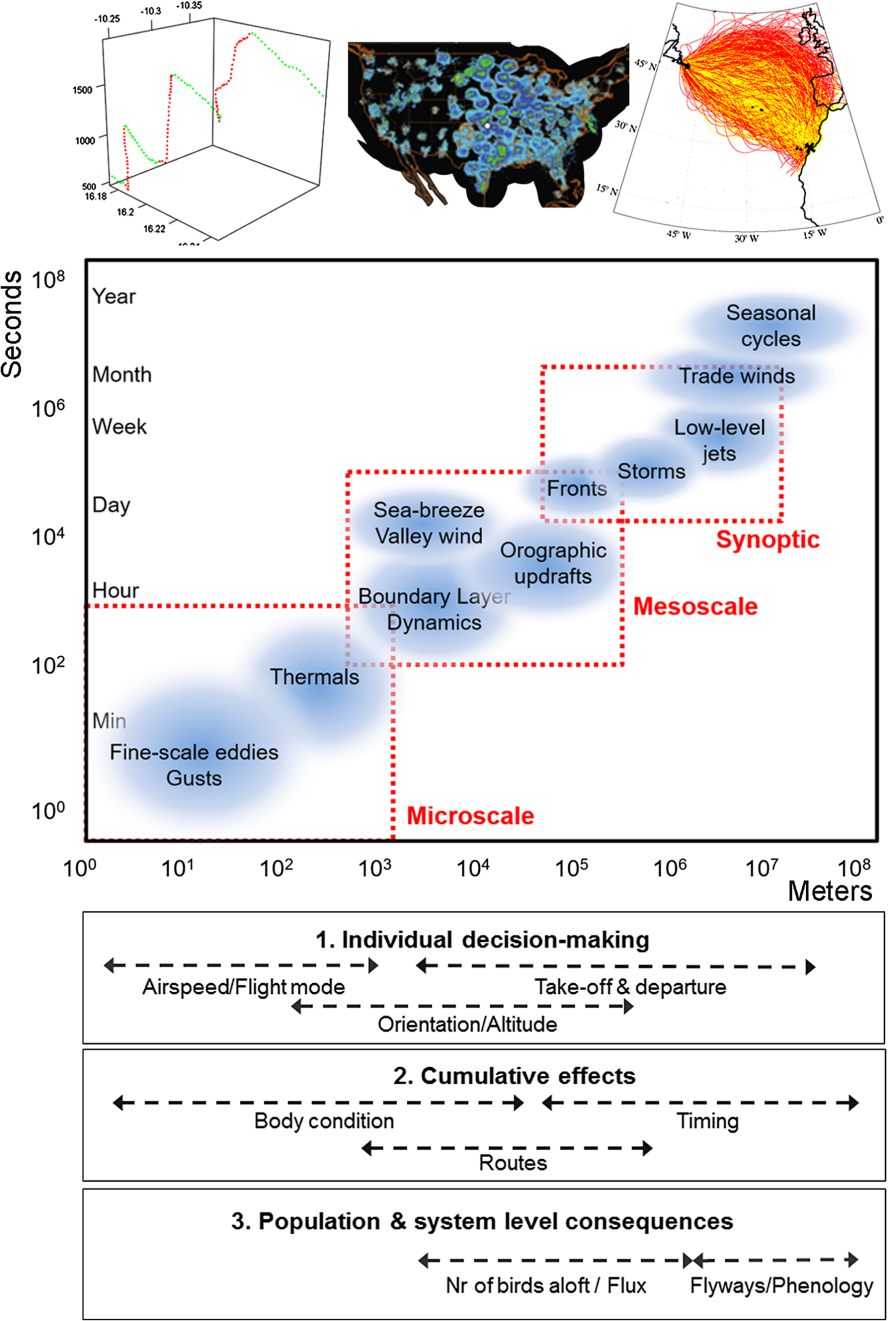
Schematic representation of atmospheric properties and processes at different scales in space (*x *axis) and time (*y *axis) that are relevant for bird migration. This review addresses (1) individual decision making in response to atmospheric conditions (e.g. alterations in airspeed, heading, flight mode or take-off decisions), (2) the cumulative effects of individual response to atmospheric conditions (e.g. impact on timing of arrival, body conditions, migration routes) and (3) population and migratory system level consequences (e.g. development of flyways, phenology). To illustrate recent advances in this field we provide three examples from recent studies in the *top* panel. Examples from *left* to *right*: biologging data used to study the fine-scale flight behaviour of soaring migrants in response to thermal convection and horizontal winds (climbing in *red*, gliding in *green*, adapted from Vansteelant et al. 2017a), weather radar networks used to study the influence of low level jets on migratory behaviour (radar reflectivity used to quantify bird migration across the United States, adapted from Wainwright et al. 2016) and mechanistic models to study the feasibility of transatlantic migration in seasonal winds (successful trajectories in *yellow* and unsuccessful trajectories in *red*, adapted from Bulte et al. (2014)

During migration, birds must contend with an environment which is highly dynamic in space and time, and they must do so efficiently. As birds can transverse hundreds to thousands of kilometres in a single flight, tens of thousands of kilometres within a migration season and perhaps hundreds of thousands of kilometres in a lifetime it may be easy to take for granted just how sophisticated the adaptations must be for dealing with such a complex environment, the aerosphere. Scientists have been studying the impact of weather on migration for decades (e.g. Smith 1917; Mackintosh 1949; Lack 1960a). For the vast majority of the 20th century studies were often limited in their spatial scope due to the technical difficulties in monitoring migratory movements. These studies were often based on visual observations (Ferguson and Ferguson 1922; Lowery 1945; Beth 1961) or local radar systems (Lack 1960b; Nisbet and Drury 1968; Bruderer 1971; Gauthreaux 1971) (Fig. 1). While visual observations may have a restricted spatial extent, constant effort sites and observation networks created opportunities for long-term studies and enabled researchers to identify recurring patterns of migration and elucidate how birds respond to weather (Allen et al. 1996; Maransky et al. 1997; Shamoun-Baranes et al. 2006). In recent decades new measurement and modelling techniques have greatly facilitated studying how birds respond to weather and what the consequences are for individuals or populations, facilitating a new age of research and discovery (Fig. 1). Biologging techniques enable researchers to study individual response to weather and other external factors along entire flyways (Fig. 2), over sea as well as over land (Gill et al. 2014; Vansteelant et al. 2015; Nourani et al. 2016; Weimerskirch et al. 2016). With high resolution GPS tracking and additional sensors such as accelerometers or heart rate monitors it is increasingly feasible to measure fine-scale changes in flight behaviour and flight energetics in response to the aerial environment (Bishop et al. 2015; Sherub et al. 2016; Vansteelant et al. 2017a). The use of operational weather radar networks, in some cases combined with citizen science based field observations, enable researchers to study migration phenology and altitudinal distribution in relation to weather over much larger areas than previously possible (Shamoun-Baranes et al. 2014; La Sorte et al. 2015b; Horton et al. 2016a). Simulation modelling and biologging have created opportunities to study potential cumulative effects of behaviour for an individual as well as population level consequences of individual behaviour (Fig. 2) (Stoddard et al. 1983; Erni et al. 2005; McLaren et al. 2012). Theoretical studies with analytical models have defined clear benchmarks or boundaries within which we expect migratory behaviour to occur generating hypotheses which can be tested in the field (Alerstam 2011; McLaren et al. 2014). Changes in data policy and ICT infrastructure have also greatly facilitated this multidisciplinary field of research as meteorological data and tools for data access and analysis are increasingly available (Shamoun-Baranes et al. 2010a; Kemp et al. 2012a; Dodge et al. 2013). The increasing diversity of research methods is translated into a rapid increase in the number of publications on weather and bird migration. Between 1995–2004 and between 2005–2014 researchers published 2–3 times more papers than in any decade during the 20th century (Fig. 1). In 2015 and 2016 already more papers have been published than were published between 1975 and 1984, or between 1985 and 1994 (Fig. 1). It is, therefore, high time to review the knowledge that we have gained over the past few decades.

Several reviews related to weather and migration already exist which cover a broad range of research (Richardson 1978; Kerlinger 1989; Alerstam 1990; Richardson 1990a, b; Liechti 2006; Shamoun-Baranes et al. 2010a; Nourani and Yamaguchi 2017). The general findings of these reviews are still what are most often conceived when considering migration and weather; for example, birds generally tend to migrate in fair weather, supporting winds and other atmospheric conditions that accompany such weather, clouds and precipitation suppress migration, soaring birds concentrate in thermal updrafts, departing several hours after sunrise as thermal convection develops and stopping before sunset and concentrating along coastlines in offshore winds. Richardson (1978) also reflected on the complexity in generalizing within or across species and distinguishing between causal and coincidental correlations.

The aim of this review is to synthesize findings in the 21st century. Current research is able to map migration routes and to quantify migratory behaviour across the globe, and to show in more detail just how migration strategies may arise, how individuals may adjust when, where and how to fly in response to atmospheric conditions and what other intrinsic or external factors may influence this response. We explore how recent studies, especially those involving techniques such as biologging, weather radar networks and mechanistic modelling (Fig. 2, top panel), have contributed to our understanding of how birds respond to weather conditions during migration, what the cumulative effects of behaviour are for an individual within the course of an annual cycle, as well as population level effects (Fig. 2) for example how weather may shape pulses of migration, population specific traits, flyways and phenology. We will consider the influence of atmospheric dynamics at different scales in space and time, from the micro-scale to global circulation patterns (Fig. 2). Understanding the flight behaviour of migrants in response to weather is also increasingly important to reduce conflicts between humans and birds within the aerosphere, for example for flight safety (van Belle et al. 2007), or the wind energy industry (Ross-Smith et al. 2016). These are very exciting times for studying the complex relationships birds have with their aerial environment during migration and we hope that this synthesis helps support and stimulate new work in this growing field of interdisciplinary research.

## Methods

This review focuses predominantly on research conducted from 2000 to 2016. We integrate studies that consider the influence of atmospheric conditions (microscale to synoptic) on migratory flight behaviour, this includes departure or landing decisions, flight altitude, flight speed, orientation strategies and flight modes. We also include theoretical studies and spatially explicit simulation models which address the impact of weather on any of the aspects mentioned above including optimal response of birds to weather conditions. We try and cover all avian taxa in this review. Topics that are excluded are mortality events or irruptive migration (Newton 2007), as well as relationships with climatic conditions that are not directly related to flight behaviour, for example phenological studies that consider long-term changes in arrival date in relation to large-scale climatic indices (Hüppop and Hüppop 2003; Usui et al. 2017).

We provide a quantitative overview of the Anglophone scientific literature during the last few decades following the criteria described above (Fig. 1). To compile the literature for Fig. 1 we conducted a systematic search from 1975 to August 2016 in Web of Science and Google Scholar. To find references before 1975 we explored references included in the (Richardson 1978) review as well as references found in Google Scholar. During literature searches the following keywords were used in different combinations: Bird*, migration, weather, meteorol*, wind, orientation, flight behaviour. The review includes online early publications and the last literature search was conducted on April 18th 2017. We only include peer reviewed journals and book chapters which include data analysis or theoretical models concerning migratory flight behaviour, excluding journals that are not listed in Science Citation Index or Scientific Journal Rankings. Reviews were not included in Fig. 1. Once the reference set was completed we attributed studies based on the main method used to the following classes: Visual (includes ringing, moon watching, ceilometer and observations from aircraft), radar, biologging, mechanistic models (sensu Bauer and Klaassen 2013) including individual based models that simulate flight behaviour, often in comparison to measurements and analytical models where data is not needed, and the category other (includes bioacoustics and experimental set ups such as in orientation cages). The full list of 366 references is available online through the Dryad data repository (Shamoun-Baranes et al. 2017).

## Atmospheric conditions—promoting or impeding migration

Meteorological phenomena (Fig. 2) occur at different scales in space and time, with some spatial and temporal dynamics that are relatively predictable and others that are more stochastic (Seinfeld and Pandis 2016). Precipitation, clouds or fog reducing visibility have long been considered as factors which temporarily suppress migration (Richardson 1978 and references therein). In this mini-review we focus on the influence of atmospheric flow on the decision-making of individual migrants and how predictable flow patterns create freeways, detours and tailbacks for migrants. In order to understand how birds cope with the heterogeneity and dynamic properties of their aerial environment, we first describe some of these properties (based predominantly on (American Meteorological Society 2016; Stull 1988; Bradbury 2000; Lutgens and Tarbuck 2010), from the perspective of a bird that has to fly through it (Fig. 2). This represents a shift from the traditional descriptions of weather in the 20th century literature, where population-scale migration phenomena were described in relation to large-scale weather phenomena dominating the weather at a specific site, to 21st century migration literature, in which we increasingly think about migratory behaviour of an individual in relation to changing weather patterns along an entire flyway.

The troposphere is the lowest layer of the atmosphere (<10 km); it is the layer in which weather is produced and birds have to cope with. Air pressure, air density and average air temperature decreases (~2 °C every 300 m) with altitude, wind speed tends to increase with altitude and nearly all clouds and moisture are confined to this lower air layer. In each hemisphere wind circulates in three cells transporting heat from tropical to polar latitudes, the Hadley cell from 0° to ~30° latitude that produces predominantly easterly winds, the trade winds, at the surface, the Ferrel cell from 30° to 60° latitude producing predominantly westerly winds at the surface, and the polar cells resulting in prevailing easterlies. The trade winds develop between 0° and 30° latitude blowing from the northeast in the northern hemisphere and from the southeast in the southern hemisphere. The intertropical convergence zone (ITCZ) is the area near the equator where these trade winds converge resulting in region of light winds and humid conditions where ascending moist and hot air creates a band of convective precipitation; the region is also referred to as the doldrums due to the prevailing weak winds. These large-scale wind circulations present over most of the earth’s surface create well-defined global circulation patterns. These may be considered the more predictable horizontal flows that are relevant for bird migration at the synoptic and global scales.

**Fig. 3:**
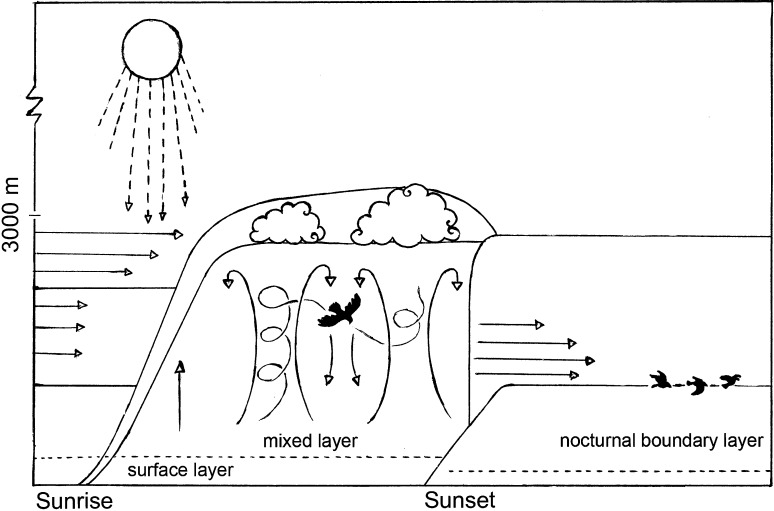
Schematic overview of the development of the boundary layer and several boundary layer properties. The *x-axis* represents time of day and the *y*-axis altitude (m). The figure shows the development of the mixed layer beginning shortly after sunrise as the sun heats the earth surface and warm air (shown as *vertical arrows*) begins to rise. As the mixed boundary layer increases in depth and vertical velocity, thermals develop (hour-glass structures). Shortly before sunset, thermals no longer form and convective turbulence decays. During the course of the night, a stable boundary layer (also called the nocturnal boundary layer) develops near the surface. Horizontal wind velocity is indicated with *horizontal arrows*. The surface layer is indicated with a *dashed line*. Drawing by Femke Lucas

Within the troposphere, it is the boundary layer, the lowest 3000 m of the troposphere that is mainly perceived by birds (Fig. 3). The boundary layer is directly influenced by the earth’s surface, and it’s properties are influenced by surface forcings such as frictional drag, evaporation and transpiration, heat transfer and changes in airflow induced by the underlying landscape at a time scale of 1 h or less (Fig. 2). Due to changes in these surface forcings, the properties of the boundary layer can vary greatly in space and in time. Boundary layer depth, for example, can vary from hundreds to thousands of meters. The surface layer is the lowest 10% of the boundary layer and is characterized by strong wind shear, with wind speed approaching zero near the ground due to surface friction and increasing logarithmically with altitude. Within the boundary layer horizontal winds of 2–10 ms^−1^ are common. The main feature that distinguishes the boundary layer from the rest of the troposphere is the relatively high occurrence of turbulence. Turbulence is responsible for vertical flow of air parcels (e.g. fine-scale eddies, gusts and thermals, Fig. 2) and is generated either mechanically by wind shear or convectively by buoyancy of air parcels.

Over land and at mid-latitudes the structure of the boundary layer is driven mainly by micro- and mesoscale processes (Fig. 2). The structure of the boundary layer is well-defined with typical diurnal oscillations that influence three main components of this structure: the mixed layer, the residual layer and the stable layer (Fig. 3). During the daytime, shortly after sunrise, a layer of well mixed air (the mixed layer) begins to develop. The mixed layer is characterized by intense vertical mixing of air generated predominantly by convection. As solar radiation heats the ground and the overlying air, warm air rises because it is less dense than surrounding air and hence positively buoyant. The resulting turbulence tends to mix heat and moisture uniformly in the vertical dimension. Due to the vertical mixing of air, the horizontal wind speed and direction are generally constant in the middle of a well-mixed boundary layer. Some parts of the earth´s surface heat more quickly than others. Warm rising air (also called convective turbulence) is often organized into identifiable structures such as thermals which scale to the depth of the boundary layer and are ~100–3000 m in diameter. Vertical velocities can reach 5 ms^−1^ or more although weaker updrafts of 1–2 ms^−1^ are more common. The increase in surface temperature due to solar heating causes the mixed boundary layer to grow in depth reaching its maximum depth in the late afternoon. Overcast skies can reduce the isolation of warm air at ground level, which in turn reduces thermal intensity. In these cases the mixed layer may exhibit slower growth and even become non-turbulent or neutrally stratified if the clouds are thick enough.

Shortly before sunset, thermals no longer form and convective turbulence decays. During the course of the night, a stable boundary layer (also called the nocturnal boundary layer) develops near the surface within which air is statically stable and tends to suppress turbulence. As the night progresses the nocturnal boundary layer gradually increases in depth. The wind profile often evolves with time during the night. Although wind at the surface frequently becomes lighter at night, winds can get considerably stronger with altitude. Under specific synoptic conditions winds at altitudes of 100–300 m above the ground (even reaching 900 m) may accelerate to very high speeds reaching 10–30 m s^−1^ in a phenomenon called a low-level or nocturnal jet (Fig. 2).

In contrast to the terrestrial environment, the sea is a highly uniform surface in terms of heat capacity, and sea surface temperatures fluctuate much more slowly in space and time than surface temperatures over land. As a result, convective turbulence is weak (at least at mid-latitudes), and boundary layer depth varies more slowly in space and time at sea than on land. Boundary layer structure is driven predominantly by mesoscale processes and under specific mesoscale conditions thermal convection can develop at sea (Woodcock 1940; Agee and Sheu 1978). Moreover, the sea is a relatively smooth surface. Surface winds are often stronger over sea than over land and a strong wind gradient develops within the surface layer.

Both vertical and horizontal flow of air can be an energetic source or sink, promoting or impeding flight during migration. For migrants that use predominantly soaring flight positive vertical flow is a source of potential energy used to gain altitude, which can be converted into kinetic energy once enough altitude had been gained (Pennycuick 2003). For birds trying to maintain a constant flight altitude, vertical flow can be an energetic sink as it potentially disrupts horizontal progress. Regardless of the mode of flight, horizontal winds affect the time and energy budget of all birds. Depending on their eco-morphology, birds differ in their flight capacity and hence their ability to exploit each of the aforementioned boundary layer structures during migration (Norberg 1990). Both over land and at sea many migratory species power migration by actively flapping their wings, for these migrants mesoscale and synoptic patterns in horizontal winds are extremely important, whereas the main importance of turbulence may be its disruption of horizontal flow. Other species, however, mainly large and heavy birds, consume too much energy while flapping to sustain an entire migratory flight. Land birds with suitable wing design can circumvent this problem by soaring in updrafts, including thermals at the microscale to orographic updrafts in which horizontal wind hits an obstacle, a mountain ridge for example, and is deflected upwards and sea-breeze fronts at the mesoscale (Fig. 2). Soaring land birds are then constrained to travel during daytime only. Seabirds with high aspect ratio wings may engage in dynamic soaring, exploiting vertical wind gradients near the sea surface that are driven by mesoscale and synoptic processes (Richardson 2011; Sachs et al. 2013).

## Individual response

### Motivation for flight

How a migratory bird responds to atmospheric conditions depends on diverse intrinsic and external factors (Nathan et al. 2008). The degree to which a migrant prioritises energy expenditure, time spent on migration or safety will also impact it’s behavioural response to atmospheric conditions (Alerstam and Lindström 1990; Jenni and Schaub 2003). The relative importance of these factors and the ability of a bird to adjust accordingly will have ecological as well as evolutionary constraints. The internal state of a migrant and external factors will also change throughout a single journey. Important intrinsic factors that may influence how birds respond to weather include the population specific endogenous program, age (experience), sex, physiological state and perhaps sociality. Similarly, body condition or timing within the annual routine may influence a bird’s motivation to fly and thus its sensitivity to weather conditions. Flight capacity defines a bird’s ability to exploit different boundary layer features. Landscape features are additional external factors that may influence how a bird responds to weather, for example responding differently to weather when travelling over what might be considered a safe or hazardous landscape (water or land), or moving towards landscape features where weather conditions supporting flight are expected. We address an individual’s response to weather from the perspective of when to fly (take-off and landing decisions), where to fly in the aerosphere (flight altitude selection) and how to fly (how speed, orientation and flight mode are adjusted in relation to horizontal and vertical flows). While the main focus is on when, where and how to fly in relation to weather, we also touch upon the intrinsic factors and landscape features that may also influence these reactions. Within each section we often distinguish between birds with different flight capacities due to its importance for how birds respond to atmospheric conditions. We distinguish between birds that use predominantly flapping flight, landbirds that use soaring flight, and seabirds that use soaring flight over oceans.

### When to fly: take-off and landing decisions

Atmospheric conditions can vary greatly within and among days, subsequently helping to power or hamper a bird’s flight. Thus, birds should fine tune their decisions on when to start and stop a migratory flight to atmospheric conditions experienced or anticipated further en route. Of all factors that make up weather, wind is a particularly important variable that has been incorporated in optimal migration models to predict take-off and stop-over decisions (Weber and Hedenström 2000). One of the reasons wind is so important is that local wind conditions, and certainly vertical changes in local wind conditions, provide information about wind conditions to be expected further ahead or later in time (Schmaljohann et al. 2011). Long periods of bad weather (e.g. precipitation) can delay departure and therefore lead to an increasing number of birds highly motivated to continue migration (tailbacks, “Zugstau”, Schüz 1952). At the first occasion of improved weather many migrants take the chance to depart, even under suboptimal wind conditions (e.g. into headwinds, Erni et al. 2002; van Belle et al. 2007).

Field observations and capture-recapture studies of passerines suggest that probability of departure from stopover sites increases with supporting winds at the surface or aloft (Schaub et al. 2004; Arizaga et al. 2011; Andueza et al. 2013; Covino et al. 2015). At ecological barriers such as the Gulf of Mexico or the Gulf of Maine, automated telemetry networks have shown that passerines adjust their departure decisions according to their body condition as well as local weather conditions, with favourable conditions resulting in a higher probability of crossing, rather than stopping or circumventing large water bodies (Deppe et al. 2015; Woodworth et al. 2015). Rain has been found to reduce the probability of departure in some studies (Schaub et al. 2004) but not all (Arizaga et al. 2011; Andueza et al. 2013). The probability of departure of migrating yellow-rumped warblers *Setophaga coronate* was shown to significantly increase with lower cloud cover (Liu and Swanson 2015) and migratory restlessness of white-throated sparrows *Zonotrichia albicollis* increased under experimentally reduced temperatures suggesting that temperature could influence departure decisions (Berchtold et al. 2017). Radar studies suggest that passerines migrating inland and at night in northwest and central Europe depart under a rather broad range of wind conditions (Erni et al. 2002; van Belle et al. 2007). However there was little migration over Central Europe in headwinds of 7 ms^−1^ or higher, such wind speeds are near the average airspeed of 10 ms^−1^ for small nocturnal migrants (Bruderer and Boldt 2001), whereas intensity of nocturnal bird migration rapidly increased in less adverse winds and in the absence of precipitation (Erni et al. 2002). Such a high tolerance for sidewinds and mild headwinds could help explain why some studies do not show a clear association between departures of passerines and wind conditions (Tsvey et al. 2007; Liu and Swanson 2015). In regions where supporting winds are infrequent and when timing of migration is constrained within the annual cycle, passerines have to select even slightly non-supporting winds on departure (Liechti and Bruderer 1998; Gauthreaux et al. 2005; Kemp et al. 2010; McLaren et al. 2012). Throughout an entire journey passerines may be flexible in how they adjust their departure decisions in response to weather; for example a study of Northern wheatears *Oenanthe oenanthe* tracked by geolocators revealed flexibility in stop-over behaviour (Schmaljohann et al. 2017). The likelihood a wheatear departed from a stop-over site increased with lower air temperature at the start of autumn migration but with higher temperature towards the end of autumn migration. In spring, the likelihood of departure and travel speeds increased more strongly with supportive winds as the season progressed (Schmaljohann et al. 2017).

Soaring landbirds, which power most of their flight with solar energy may be more selective for precipitation, cloud cover and convective conditions than horizontal wind flow. In ospreys *Pandion haliaetus*, which use a combination of flapping and soaring flight, departure and landing decisions were not related to wind conditions (Thorup et al. 2006), possibly because their low flight costs, which makes resting without refuelling unattractive. Thorup et al. (2006) did not find an effect of rainfall on stop-over decisions. This may be because the spatial–temporal resolution of global rainfall data is still too coarse to enable reliable path annotation, but also because stop-over behaviour may be very flexible and therefore challenging to describe and quantify based on the environmental conditions during the days of migration of just a few tracked individuals. Anno 2017, already much larger tracking datasets have become available. A recent study of Montagu´s harriers *Circus pygargus* showed the birds travelled faster and more hours per day with increasing tailwinds, yet they interrupted travel in strong headwinds, even while crossing the Sahara (Klaassen et al. 2017).

Timing migration with supporting winds may be especially important for species or populations engaging in long-distance non-stop flights. For example, red knots *Calidris canutus* have been observed to delay departure from a stopover site for several hours before engaging in a non-stop flight of several thousand kilometres and then leave en masse once a storm front passed (Leyrer et al. 2009). At an important stopover area in the Yellow Sea, shorebirds departed in predominantly supporting winds at low altitudes in spring and autumn (Ma et al. 2011). Individual-based models as well as individual tracking studies reveal that departing on days when wind conditions are favourable, can be essential for survival for passerines as well as waders crossing ecological barriers such as oceans (Pennycuick and Battley 2003; Bulte et al. 2014; Gill et al. 2014). Waiting for the right conditions for departure can convert an atmospheric barrier into a freeway for fast and efficient flight (Felicísimo et al. 2008; González-Solís et al. 2009; Bulte et al. 2014), especially when wind conditions at departure are indicative of conditions further en route. Nevertheless, observations of bar-tailed godwits *Limosa lapponica* that depart from New Zealand on a long transoceanic flight to SE Asia in spring revealed these individuals did not delay annual departure to select the most profitable winds, yet some individuals did advance their departure date in years when favourable winds were unusually early (Conklin and Battley 2011). Whimbrels *Numenius phaeopus* that cross the Atlantic in a non-stop flight from West Africa to Iceland start migrating in headwinds in spring, while they usually depart in tailwinds during autumn (Alves et al. 2016). It could be that arctic breeding birds such as godwits and whimbrels risk transoceanic flights in headwinds instead of waiting for tailwinds because of high rewards for early arrivals at the breeding sites and low probability of supporting winds at departure.

How selective birds are for suitable flight conditions will depend on the time constraints within the annual cycle (defining how long a bird can afford to wait for suitable conditions), the physiological condition of the bird and the temporal distribution of suitable conditions (how long does a bird have to wait) and balancing the trade-off between the time spent waiting and the time or energy gained during flying in favourable conditions (Alerstam and Lindström 1990; Weber and Hedenström 2000; Gauthreaux et al. 2005). Similarly, the onset of migration may be influenced by environmental conditions at different scales in space and time, with seasonal environmental cues at the larger spatio-temporal scale influencing general readiness for migration and selectivity of favourable environmental conditions influencing finer scale take-off decisions. As studies are still quite scattered across species, regions and scales of analysis, integration of methods and comparative analysis are needed to reveal general rules in the take-off and landing decisions of migrant birds in relation to weather. As more species and larger numbers of individuals are individually tracked along their entire migration routes, this should become increasingly feasible.

### Where to fly: choosing the right altitude

Atmospheric conditions can differ greatly with altitude and differences between conditions very close to the earth surface (up to tens of meters) and at upper altitudes may have a direct impact on flight energetics (e.g. wind speed, air density, vertical updraft velocity), safety (e.g. cloud cover, fog, rain), or eco-physiology (e.g. temperature, humidity). Thus birds, can control the atmospheric conditions they experience by selecting specific flight altitudes, this within realistic boundaries in which the benefits of changing flight altitude outweigh the costs. Based on optimal migration theory, birds using flapping flight are expected to fly at altitudes with winds that enable them to maximize their speed along their preferred track direction. This behaviour may result in a change in altitude during a single flight depending on the wind conditions experienced (Alerstam 1979). Flight altitude is technically challenging to measure, especially with high flying migrants, thus tools such as radar and biologging have been instrumental in studying how birds adjust their flight altitudes in response to weather conditions. These studies have shown flight altitude can vary greatly and that birds are generally concentrated within the first few hundred meters of the boundary layer, yet flight altitudes can also reach altitudes of several kilometres above the earth surface (Liechti and Schaller 1999; Shamoun-Baranes et al. 2003b; Schmaljohann et al. 2009; Kemp et al. 2013; La Sorte et al. 2015b).

Broad-front nocturnal migrants, predominantly passerines, migrating over land in the trade-wind zones, select flight altitudes with supporting winds whereas other factors such as temperature and humidity are less influential (Bruderer et al. 1995; Liechti et al. 2000; Liechti and Schmaljohann 2007; Schmaljohann et al. 2009; Horton et al. 2016b). In these regions flight altitude selection is driven predominantly by the energetic cost of flight rather than other physiological costs such as their water balance (Liechti et al. 2000; Schmaljohann et al. 2009). In the mid-latitudes weather radar studies have shown that birds generally increased their flight altitude to select supporting winds when winds at the surface were prohibitive and climbed to altitudes where wind was acceptable but not necessarily optimal (Dokter et al. 2013; Kemp et al. 2013). Specific synoptic conditions, such as the presence of low-level jets (Fig. 2) (La Sorte et al. 2015b; Wainwright et al. 2016), or the passage of high-pressure systems (Dokter et al. 2013) can create prohibitive winds in one season and supporting winds in another or prohibitive winds at the surface and supporting winds at higher altitudes. Consequently these wind patterns result in high densities of migrants concentrating in layers at high altitude in one season but not in another due to the spatio-temporal configuration of these synoptic conditions in relation to the prevailing migration direction. While radar studies can be used to compare altitude distributions in space and time, we know very little about what an individual bird does during the course of a single flight, especially for passerines. One exception is a recent study on migrating Swainson’s thrushes *Catharus ustulatus* in North America (Bowlin et al. 2015). Birds were equipped with transmitters that recorded pressure and temperature and birds were tracked by aircraft to retrieve data. Excluding initial ascent and final descent of each flight this study showed intriguing and significant (>100 m) changes in flight altitude during the course of a single night. This suggests that while passerines may select flight altitudes based on wind conditions they may not always maintain constant flight altitude within a single flight.

Numerous other species groups that use flapping flight, including small raptors, waterbirds and seabirds have also been shown to adjust their flight altitude in response to weather conditions (Kerlinger and Gauthreaux 1984; Krüger and Garthe 2001; Mateos-Rodríguez and Liechti 2012; Kahlert et al. 2012). There seems to be a general consensus that flapping flight of seabirds at sea, though not necessarily during migration, occurs at relatively low flight altitudes <100 m (Garthe and Hüppop 2004; Ross-Smith et al. 2016). Within this relatively narrow altitude band, studies have shown that as expected from optimal migration theory seabirds as well as waterbirds fly at higher altitudes with supporting winds and lower altitudes with opposing winds (Krüger and Garthe 2001; Kahlert et al. 2012; McLaren et al. 2016) with waterbirds also reducing their flight altitude with increasing wind speed (Kahlert et al. 2012). Visibility also influenced flight altitude, but predominantly at night (Kahlert et al. 2012). However, as with nocturnal migrants, diurnal migrants may not select the flight altitude with the most supportive winds but a lower local optimum (Mateos-Rodríguez and Liechti 2012). Similarly studies of geese (Hawkes et al. 2013) and swans (Klaassen et al. 2004) have shown that while birds alter flight altitudes during flight, birds seem to generally prefer staying within a few hundred meters of the surface and do not select altitudes with the most supporting winds, likely due to the mechanical and physiological cost of climbing which outweigh the potential benefits of selecting supporting winds.

For species that use predominantly soaring flight during migration, flight altitude selection depends on the type of uplift used. When utilizing thermal convection, birds will climb in thermals gaining altitude and then glide between thermals, making forward progress towards their subsequent destination. Studies have shown that the maximum altitude during the climbing phase, or mean flight altitudes (when climbing and gliding cannot be discerned) are positively influenced by thermal depth (the depth of the boundary layer) and thermal uplift velocity or proxies thereof (Shannon et al. 2002; Shamoun-Baranes et al. 2003b; Chevallier et al. 2010; Bohrer et al. 2012; Mellone et al. 2015). Thus, the deeper and stronger the thermals, the higher birds will climb before leaving a thermal to glide to the next thermal. Flight altitudes during migration follow similar patterns, increasing gradually during the morning as the convective boundary layer develops, reaching a peak during midday and decreasing abruptly before dusk (Fig. 3). Birds will generally leave weak thermals earlier than strong thermals. In areas with well-developed convective conditions birds will regularly climb to flight altitudes of thousands of meters, whereas in areas with weaker convective conditions birds may only climb to altitudes of several hundred meters. Among species, the depth of the boundary layer utilized during flight is strongly influenced by the flight capacity of the species (Shamoun-Baranes et al. 2003b). Soaring migrants may also use orographic updrafts to power soaring flight but must stay relatively close to the source of deflection and thus do not fly to altitudes reaching hundreds or thousands of meters above the surface (Bohrer et al. 2012; Duerr et al. 2015; Katzner et al. 2015). At sea, thermally induced convective flow can develop under specific air-sea temperature differences and horizontal winds speeds at the surface (Woodcock 1940; Agee and Sheu 1978). The use of thermals at sea was noted decades ago by (Woodcock 1940) in herring gulls *Larus argentatus*. More recently, it has been shown that great frigate birds *Fregata minor* can travel hundreds of kilometres using convective soaring, climbing predominantly to altitudes of 500–600 m but occasionally also climbing quickly to altitudes of 1000–2000 m (4120 m maximum altitude) (Weimerskirch et al. 2016). In addition to using orographic and convective lift to gain altitude during soaring flight, several species of seabirds (especially those with a high aspect ratio) flying very close to the sea surface to take advantage of the logarithmic change in wind speeds with altitude to power dynamic-soaring (Richardson 2011; Sachs et al. 2013).

### How fast to fly in relation to the weather

The airspeed of a migrant bird, i.e. its speed relative to the surrounding air, has important ramifications for the rate at which a bird consumes energy during flight. A flapping bird powers flight by burning endogenous energy stores deposited as fat and protein, while soaring birds are solar-powered and vertical wind-powered flyers. Regardless of what form of energy is used to power flight, a trade-off exists between a bird’s airspeed and the rate at which energy is consumed. Optimal migration theory incorporating the effect of wind or thermal convection has been used to model optimal airspeeds for minimizing flight time or energy expenditure under different atmospheric conditions (Liechti et al. 1994; Pennycuick 2008; McLaren et al. 2014) and often used as a theoretical benchmark for comparison with field observations. For example, when flying at maximum range airspeed, the airspeed at which energy expenditure per unit distance travelled is minimized, a bird should reduce its airspeed in tailwinds and increase its airspeed in side- and headwinds, depending also on the extent to which it compensates for wind drift (Liechti et al. 1994) (see section “How to fly: adjusting orientation strategies in response to horizontal and vertical flows”). Numerous studies have shown that migrants using flapping flight increase their airspeed in headwinds, however analysis and interpretation is occasionally hampered by the method used to quantify supporting winds (Shamoun-Baranes et al. 2007). Coastal observations indicate seabirds mostly decrease airspeed with tailwinds and increase airspeed in headwinds, similar to landbirds (Mateos-Rodríguez and Bruderer 2012). Biologging has shown that during sea crossings, lesser black-backed gulls *Larus fuscus* increase their airspeed during flapping flight in headwinds and cross winds, but still fly slower than optimal for minimizing energy expenditure (McLaren et al. 2016).

While not studying adjustments of airspeed in relation to weather directly, numerous biologging studies have shown that a broad range of soaring migrants over land achieve higher hourly or daily ground speeds under better convective conditions and with supporting horizontal flow (Mandel et al. 2008; Chevallier et al. 2010; Mellone et al. 2015). Several studies show that the seasonal and regional differences in the hourly or daily ground speeds of soaring migrants are strongly influenced by atmospheric conditions (Shamoun-Baranes et al. 2003a; Mellone et al. 2012, 2015; Vansteelant et al. 2015; Rus et al. 2017), rather than by seasonal differences in the motivation to minimize travel time. How soaring migrants adjust their airspeeds to weather conditions is less clear. While increasing airspeeds while gliding can reduce the time spent in flight it can also increase the risk of reaching the ground or having to transition to flapping flight before reaching the next source of uplift. Thus soaring birds can afford to glide at higher airspeeds under well-developed convective conditions. Radar studies have shown that in several species that use convective soaring, birds increase their gliding airspeed with increasing climb rates in thermals (Kerlinger 1989; Spaar and Bruderer 1996; Spaar 1997), suggesting they adjust their airspeed in response to atmospheric conditions to minimize flight time. More recently, it has been shown that large soaring birds have a tendency to maximize gliding range (distance travelled over the surface) rather than minimizing flight time when soaring at low altitude (Horvitz et al. 2014; Harel et al. 2016). To maximize the range during every glide, thermal-soaring birds can adjust their airspeed in relation to wind. High-resolution tracking of soaring migrants revealed that honey buzzards *Pernis apivorus* for example adjust gliding airspeed to maximize gliding range more than minimizing travel time under poor soaring conditions (Vansteelant et al. 2017a). Differences in prevailing soaring and wind conditions between geographical regions and across seasons thus lead to the emergence of highly flexible airspeed adjustments in relation to weather (Vansteelant et al. 2017a).

Studies across a range of taxa and flight modes show that birds fly at slightly higher airspeeds than predicted to conserve energy, although they still reduce airspeed in tailwinds and increase airspeed in headwinds. The predicted relationship between energy expenditure and airspeed is rather shallow near maximum-range airspeed. Therefore, birds can increase their airspeed by a few ms^−1^ above theoretical maximum-range airspeed, without a large energy investment. Nevertheless, to further increase migration speed it may be more beneficial to make the most of prevailing atmospheric circulation patterns by adjusting headings rather than further increasing airspeed (McLaren et al. 2016). In addition to flexibility in the bird´s motivation to adjust airspeed, some of the conflicting evidence about airspeed adjustments from site-specific studies can potentially be explained by environmental factors that influence a bird’s ability to adjust or accurately assess their air speed. Mismatches between theoretical model predictions and field measurements may also be due, in part, to limitations of the theoretical models used to develop predictions.

### How to fly: adjusting orientation strategies in response to horizontal flows

Assuming the goal destination of a migrant is known, we can determine its orientation behaviour in relation to wind or other atmospheric conditions that function as an energetic source or sink for migratory flight. For an individual migrant, orientation behaviour in response to weather may vary depending on the motivation for flight, its navigational capacity and past experience, topography, availability of navigational cues and experience. Orientation strategies in relation to wind has received quite some attention in the past from a theoretical as well an empirical perspective (Liechti 2006). An optimal orientation strategy in variable winds often combines different orientation behaviours within the course of a single flight, which can result in longer but faster migration routes (McLaren et al. 2014).

Radar studies from Europe and North America have shown that passerines that migrate at night and overland tend to compensate more for sidewinds during spring than autumn (Bäckman and Alerstam 2003; Horton et al. 2016a). While a local radar study in Sweden showed that common swifts *Apus apus* compensated equally for sidewinds during autumn and spring migration (Karlsson et al. 2010). While birds not compensating during flight may end up off course, they also have strategies for dealing with displacement due to wind. For example, a wide range of passerine species migrating over eastern North America engage in morning flights from coastal sites in which they compensate for wind drift after nocturnal flights in strong sidewinds (Van Doren et al. 2016). Individual based models have shown that for migrants expected to have a limited navigational capacity, for example juvenile passerines, partial drift (McLaren et al. 2012) and weak selectivity for supporting winds would result in a feasible migration strategy. For autumn in western Europe where opposing winds prevail even full drift with endogenous headings that are adaptively evolved to prevailing winds would be a feasible strategy (Erni et al. 2005). In contrast, some species may fully compensate for sidewinds, as has been shown for European bee-eaters *Merops apiaster* migrating over southern Israel, regardless of whether they migrated by flapping or soaring (Sapir et al. 2014). Studies have also shown that birds orient differently in relation to wind over land compared to over sea or along coastlines. For example, passerines were found to compensate more for sidewinds along coastlines compared to inland (Horton et al. 2016a). Arctic breeding waders on autumn migration through southern Sweden tend to drift with sidewinds as they fly over the Baltic Sea and start compensating once they fly over land at high altitude (Grönroos et al. 2013).

In studies using radar or visual observations the intended direction of migrants is often estimated using the mean heading or travel direction observed at that site, unless a more concrete destination can be assumed. This has its limitations as individuals with different preferred directions may cross a radar at the same time (Green and Alerstam 2002; Kemp et al. 2012b). With biologging, a bird’s orientation behaviour relative to individually consistent destinations (Chapman et al. 2011; Kemp et al. 2012b) can be quantified along an entire flyway. Orientation behaviour has been quantified in this way for several species of facultative and obligate soaring raptors. The results revealed that several species have highly flexible orientation behaviour, including distinct regional and seasonal orientation strategies to cope with prevailing winds (Klaassen et al. 2011; Liminana et al. 2013; Vidal-Mateo et al. 2016). Honey buzzards even migrate against the direction of prevailing winds in tropical West Africa at the start of spring migration, in order to use a corridor of favourable winds to cross the Sahara later on their journey. After crossing the Sahara they overcompensate for sidewinds to reach the Straits of Gibraltar (Vansteelant et al. 2017b). These birds do not follow the same route every year. They change travel direction to start the desert-crossing at a different point, depending on where the winds become favourable. In a similar fashion, Eleonora’s falcons *Falco eleonorae* cross the Strait of Mozambique, between Madagascar and eastern continental Africa at a different location every year, but nearly always where winds were most supportive (Mellone et al. 2011). Studies of golden eagles *Aquila chrysaetos* and turkey vultures *Cathartes aura* in North America have shown how individuals will adjust their routes to fly along mountain ridges when orographic lift is available (Bohrer et al. 2012; Duerr et al. 2015; Katzner et al. 2015). Despite this high flexibility in route choice, seasonal differences in prevailing winds relative to the travel direction of migrant birds mould distinct flyways between autumn and spring at individual and population level. Besides landbirds, also soaring seabirds, such as albatrosses, shearwaters and terns, have been tracked along migration routes that are aligned with prevailing atmospheric circulation patterns (Shaffer et al. 2006; González-Solís et al. 2009; Egevang et al. 2010; Dias et al. 2013). Interestingly, such complex migration routes and the underlying orientation strategies are not inherited genetically in terrestrial soaring migrants (Hake et al. 2003; Thorup et al. 2003) while juvenile albatrosses do seem able to locate the most suitable flyways and non-breeding areas using an innate migration program (Åkesson and Weimerskirch 2005). Juvenile migrant landbirds instead drift with prevailing winds towards an unknown location on their first migration (Thorup et al. 2003; Rus et al. 2017) and survivors tend to return near their natal site for breeding once adult. In such systems there is great potential for predictable global atmospheric circulation patterns to shape not only migration flyways but also the distributions of species, especially during the non-breeding season (Cresswell 2014).

### How to fly: choosing the right flight mode

Flight kinematics and hence flight modes may change along a continuum. The mode of flight a bird uses influences what defines supporting or prohibitive flight conditions and the way it can respond to weather. Although we generally treated flapping, terrestrial soaring and soaring seabirds separately, there are species that are able to switch between different flight modes depending on atmospheric conditions. One of the main challenges is the ability to measure flight mode and wing beat kinematics during migration. Tracking radar has been used for short distances to show how birds adjust flight mode (Spaar and Bruderer 1997) to atmospheric conditions and flight kinematics to air density (Schmaljohann and Liechti 2009). Biologging, specifically accelerometers, can be used to identify flight mode as well as flight kinematics for entire migration journeys (Liechti et al. 2013; Hedenström et al. 2016; Rotics et al. 2016; Shamoun-Baranes et al. 2016a). Similarly, flight mode can be inferred from high resolution measurements and changes in speed, direction and altitude (Treep et al. 2016; Vansteelant et al. 2017a).

Large soaring migrants travelling along mountain ridges which are aligned along the primary axis of migration may switch from convective soaring to slope soaring utilizing orographic lift, especially when convective conditions were weak or wind speeds are high (Duerr et al. 2012; Lanzone et al. 2012; Katzner et al. 2015). At times, obligate soaring migrants must support migration by flapping during parts of their journeys, especially when making extended flights across water bodies in the absence of thermal convection (Meyer et al. 2000). Other species travel predominantly by flapping and facultatively engage in thermal soaring to reduce energy expenditure (Spaar and Bruderer 1997). Facultative migrants may extend their flight to late evening or starting early in the morning by using flapping flight (Stark and Liechti 1993) when convective conditions are not developed enough to support soaring. A surprisingly small terrestrial migrant which adjusts its flight mode to weather conditions during migration is the European bee-eater (Sapir et al. 2011a, b), with the propensity to use soaring flight increasing as conditions supporting thermal convection improved. At sea, a range of seabirds that use flap-gliding, increase their propensity for using dynamic soaring with higher winds speeds (Ainley et al. 2015). Gulls flying over land and sea are true flight generalists that rapidly respond to changes in the atmospheric environment in order to optimize flight performance using predominantly flapping flight and flap-gliding but with well mixed boundary layer conditions, or when orographic lift is available they switch to soaring flight (Woodcock 1940; Shamoun-Baranes et al. 2016a).

## Cumulative effects of individual behaviour

Atmospheric conditions have a strong impact on individual decisions influencing when, where and how to fly during migration. These decisions accumulated over time have consequences for the timing, the migratory route, the area of arrival, the energy budget, the physiological condition and finally the individual’s survival and reproductive success (Fig. 2). These cumulative effects can be considered within the course of a single flight, an entire migratory journey, the annual cycle through carry-over effects across phases in the annual cycle (Harrison et al. 2011) or even across different life-stages through ontogenetic effects (Senner et al. 2015b). Mortality is an extreme consequence of an individual’s response to weather conditions. Although there are numerous reports or suggestions of weather related mortality, generally, little is known of the bird’s behaviour, making it difficult to directly link individual decision making to these mortality events (Newton 2007; Strandberg et al. 2010; Diehl et al. 2014; Lok et al. 2015). Most studies that explicitly incorporate the response of birds to local weather conditions consider cumulative effects within the course of a single flight or entire migratory journey. It is not difficult to envisage the cumulative effects of individual response to weather, for example, weather may delay the onset of migration, reduce travel speeds en route, delaying arrival times at the breeding grounds or reduce a bird’s physiological condition on arrival, subsequently reducing breeding success through carry-over effects (Drake et al. 2014).

Most biologging studies of migrant birds report travel distances and ground speeds at which birds travelled as measures of migratory performance and hence a cumulative effect of behaviour en route. However, these ground speeds are strongly influenced by weather. For example, honey buzzards and Montagu’s harriers achieve higher hourly speeds and daily travel distances in spring than in autumn and over the Sahara compared to Europe, but because of differences in weather conditions rather than motivation (Vansteelant et al. 2015). In North-America, juvenile golden eagles achieve higher travel speeds than adults, but mainly because they depart later in stronger winds and travel downwind more than adults (Rus et al. 2017). We strongly recommend that wind should be accounted for when using ground speeds as a measure of migratory performance, especially when weather conditions experienced are expected to during migration, within or among the species of interest. Mechanistic models can provide insight into the consequence of cumulative effects, such as delayed arrival at breeding sites, without necessarily focusing on the mechanism causing the effects (Kokko 1999). Spatially explicit simulations have been particularly helpful in showing the cumulative impact of individual response to weather conditions at departure and en route. Different behavioural adaptations can be tested in the same study system (e.g. departure decisions, flight altitude, orientation behaviour), behavioural responses can be kept constant while only the environmental conditions change, and some behavioural responses can be kept constant while others may change, enabling researchers to disentangle possible concomitant adjustments of behaviour in response to weather. Such studies have shown how selecting when to fly (e.g. departure date, or wind conditions at departure) can have a major impact on flight duration, energetic cost of flight and even on survival, especially when crossing ecological barriers (Shamoun-Baranes et al. 2010b; McLaren et al. 2012; Bulte et al. 2014). Numerous models have shown just how influential the selection of flight altitude can be (Stoddard et al. 1983; Erni et al. 2005; Gauthreaux et al. 2006; Shamoun-Baranes and van Gasteren 2011; McLaren et al. 2012; Bulte et al. 2014; McLaren et al. 2014). For non-stop flights across ecological barriers, selecting the right flight altitude can be the difference between life and death for a small passerine (Erni et al. 2005; Bulte et al. 2014). Similarly, wind conditions may differ so much at different altitudes that a 4000 km non-stop flight of red knots can vary between 3 and 7 days at the same departure date and time and depending only on the flight altitude selected (Shamoun-Baranes et al. 2010b). Similarly, models have shown that birds maintaining constant orientation behaviour in relation to wind may not always reach their destinations on time, run out of fuel, or be blown far off course (Erni et al. 2005; McLaren et al. 2012; Bulte et al. 2014; McLaren et al. 2014). The energetic consequences of flight behaviour in response to weather can also be measured using biologging techniques that incorporate tracking as well as heart rate monitoring or proxies of energy expenditure based on body acceleration. Studies across taxa have shown how much energy is saved by utilizing soaring flight when atmospheric conditions are supportive (Mandel et al. 2008; Sapir et al. 2010, 2011b; Weimerskirch et al. 2016). The capacity to study energetic consequences of flight behaviour are likely to increase significantly in the coming years.

As studies are integrating different sources of information, carry-over effects of a bird’s response to extreme weather events can be investigated; for example, (Senner et al. 2015a) found that despite delayed and even reverse migration during an extreme cold event during spring migration, there was little impact on breeding success of continental black-tailed godwits *Limosa limosa*. Increasingly, studies are showing that individuals may be rather flexible in their migratory behaviour, altering their response to weather en route. This may be by switching their flight mode as sources of lift change, altering their orientation strategies, fine tuning their time of departure in response to extreme weather events (Leyrer et al. 2009; Lanzone et al. 2012; Miller et al. 2016). Flexible response to weather may buffer the cumulative effects that would have otherwise been incurred had a bird maintained a rigid behavioural response to weather conditions. An individual’s ability to respond flexibly to weather conditions may very well depend on flight capacity or experience as shown in several biologging studies (Miller et al. 2016; Rotics et al. 2016; Rus et al. 2017) and on factors that affect the ability of a migrant to sense or gauge its own airspeed, heading and altitude (Hedenström and Åkesson 2017). Thus, in order to cope efficiently with the heterogeneity of weather conditions an individual might experience, the evolution of behavioural plasticity may be more favourable (Mery and Burns 2010) than the evolution of fixed reaction norms [e.g. behavioural syndromes (Sih et al. 2004)].

## Population and migration system level consequences

When migratory populations converge in space and time at the scale of hundreds kilometres and within days or weeks of each other they can be exposed to similar synoptic and mesoscale weather systems (Fig. 2). While individuals may adjust their flight behaviour to conditions encountered en route, they are also likely to respond to similar weather conditions in the same landscape in roughly the same way. This is most evident under extremely adverse weather, which can lead to emergency stop-overs by large numbers of migrants (Shamoun-Baranes et al. 2010b; Overdijk and Navedo 2012). The arrival of fair weather after prolonged periods of suboptimal flight conditions, on the other hand, can spur mass migration events. This is true at geographical bottlenecks and leading lines for migrant birds as well as more inland migration sites (Allen et al. 1996; Shamoun-Baranes et al. 2006). The ability to predict migration fluxes of a range of nocturnal migrants based on local weather (Erni et al. 2002; van Belle et al. 2007) strongly supports the proposition that birds will have similar responses to weather within certain periods of time and parts of a flyway (Farnsworth et al. 2016). Differences in flight capacity of different species, for example due to body mass and wing shape, are probably most influential in shaping species-specific flight behaviour and route choice at local (Vansteelant et al. 2014), regional (Panuccio et al. 2017) and flyway-scale.

Migration flyways are one of the most prominent population level consequences of individual response to weather conditions (Alerstam 2001; Bildstein and Zalles 2005). Many migratory species, whether they be passerines, waders, raptors or seabirds, make a loop migration, some travelling across or between continents in circuitous patterns that differ greatly between spring and autumn (Meyburg et al. 2003; Egevang et al. 2010; Battley et al. 2012; Tøttrup et al. 2012). These contrasting seasonal patterns have often been suggested as being driven by weather (Moreau 1972; Elkins 2004) and recent studies are providing increasing support for several systems. In the eastern North American flyway the loop migration of passerines is shaped by the US Great Plains Low Level Jet that generates tailwinds for migrants in spring, but headwinds in autumn (La Sorte et al. 2014; Wainwright et al. 2016). Individual tracking revealed that adult honey buzzards make a westward detour of West Africa in spring to catch a tailwind across the Sahara en route to the Straits of Gibraltar and into western Europe (Vansteelant et al. 2017b). By travelling further west in spring than in autumn, a loop migration arises that resembles that of other migrants, including marsh harriers *Circus aeruginosus* (Klaassen et al. 2010b). Entire populations of waders simultaneously cross the Pacific Ocean between Alaska and New Zealand relying on predictable and supportive winds, circumventing this region during spring migration when they return to their breeding grounds (Gill et al. 2014). Oriental honey buzzards *Pernis ptilorhynchus*, migrants that rely on convective soaring, make remarkable sea crossings in autumn, relying on supporting winds and seasonal convection and detours in spring when weather conditions do not support soaring over sea (Yamaguchi et al. 2012; Nourani et al. 2016). Seabirds also wander the oceans by repeating looped circuits aligned with favourable winds that enable them to soar effortlessly for months on end (González-Solís et al. 2009; Kopp et al. 2011). In general we expect that global atmospheric circulation patterns cause bird migration flyways to converge in space and time across a wide range of migratory taxa. On a global scale, simulation models reveal that the most efficient global aerial flyways do not follow constant geomagnetic or geographic course, but instead they are shaped by synoptic and mesoscale atmospheric processes (Kranstauber et al. 2015). Winds may not only shape migratory flyways and freeways, but also global distribution patterns of migrants (Suryan et al. 2008; Weimerskirch et al. 2012; Cresswell 2014). Through adaptive evolution, prevailing winds and underlying landscape may shape flight related traits (Fig. 2) such as endogenous headings and wind selectivity (Erni et al. 2005; Gauthreaux et al. 2005; Vrugt et al. 2007).

Individual response to weather may influence population dynamics directly through mass mortality events or indirectly through the impact of weather on phenology or physiological state with subsequent consequences for breeding success. However, these feedback loops between individual behaviour and population dynamics have not been investigated in detail. There are numerous reports of mass mortality events among migrants, especially landbirds that encountered a bad weather spell while crossing open water (Newton 2007; Diehl et al. 2014). Such events can be of such a magnitude that they are likely to impact on population dynamics (Newton 2006, 2007; Ellis et al. 2016). While theoretical and empirical studies have shown how changes in migration timing can influence population dynamics, for example, through earlier arrival at the breeding grounds, a link to the behavioural mechanisms that would result in subsequent phenological changes is generally lacking (Kokko 1999; Knudsen et al. 2011).

## Concluding remarks

Biologging studies have contributed greatly to our understanding of how individuals respond to microscale through mesoscale conditions (Fig. 2). They have been instrumental in revealing the behaviour plasticity of individuals in their respond to atmospheric conditions, perhaps one of the most striking findings across studies of different taxa (Klaassen et al. 2010a; Conklin and Battley 2011; Mellone et al. 2011; Sapir et al. 2011b; Horvitz et al. 2014; Vansteelant et al. 2017b). Generalisations often remain elusive as studies increasingly reveal the complex interactions between intrinsic and external factors that drive how birds respond to weather. Independent studies often focus on one aspect of behaviour, but considered together, it becomes clear that an individual can use different strategies for dealing with environmental heterogeneity (altering departure decisions, air speeds, orientation strategies, flight altitudes). This may enable individuals to buffer the negative cumulative effects that could arise from a rigid response to environmental conditions experienced en route. Some biologging studies have revealed how fine-scale behaviour and larger scale atmospheric phenomena can shape migratory flyways and phenology. While biologging studies provide the individual’s perspective, radar studies have provided a more synoptic perspective showing how meso- and synoptic scale conditions and landscape features experienced simultaneously by a range of species can influence migratory behaviour similarly. In the future, we expect information on individual behaviour gained from biologging studies to be combined with radar data to extrapolate individual behaviour to larger scale processes on the population level. Integrating such combined results into mechanistic models will allow to explore the consequences of behaviour in dynamic environments at the individual as well as population level.

Comparative analysis within and among species will be crucial for identifying leading selective pressures and evolutionary constraints that shape individual response to atmospheric conditions, convergent migration strategies, as well as population level consequences of behaviour. Comparative analysis is becoming increasingly feasible due to improved infrastructure and awareness for the storage and exchange of data as well as analysis and visualization tools, enabling researchers to bring together information across multiple studies and disciplines (Kemp et al. 2012a; Dodge et al. 2013; Shamoun-Baranes et al. 2016b). The increasing use of accelerometers, heart-rate monitors and other eco-physiological sensors will enable us to understand the energetic consequences of flight behaviour in response to weather in a broader range of species and throughout the annual routine (Sapir et al. 2010; Rotics et al. 2016; Weimerskirch et al. 2016). As tracking devices become smaller, cheaper and more durable, we can extend the sample sizes and range of taxa that can be studied as well track individuals for several years improving our capacity to sample migratory behaviour under a broad range of atmospheric conditions, including rare events.

One of the potential limitations in studies of fine-scale flight behaviour during migration (Fig. 2, lower left corner) is the current mismatch in the resolution of avian tracking and meteorological data (Shamoun-Baranes et al. 2010a). Meteorological datasets are generally available at a spatial and temporal resolution which is much lower than movement data collected with high-resolution GPS tracking. While such meteorological data still provide a good reflection of mesoscale and synoptic patterns (Fig. 2, upper right corner) higher-resolution weather models are needed to estimate fine-scale conditions experienced by birds in very heterogeneous and dynamic environments (Shannon et al. 2002, Shamoun-Baranes et al. 2003b; Sapir et al. 2011a; Harel et al. 2016). Although it may take some time before such high-resolution weather data can readily be generated across large spatial domains covering entire migration flyways, fine-scale environmental conditions that migrants encounter along the way can already be estimated by using tracking information in innovative ways. For example, wind conditions can be estimated along a bird’s flight path from tracking data directly (Treep et al. 2016; Weinzierl et al. 2016; Yonehara et al. 2016).

Tracking individuals from fledging to adulthood will make important contributions to our understanding of how weather influences the development of individual migration routes and potentially the evolution of flyways in response to weather. Such tracking studies are just beginning to unravel how flight and orientation capacity of migrant birds develop with age or social learning (Mueller et al. 2013; Sergio et al. 2014; Harel et al. 2016), and what mechanisms lead to the development of conservative as well as flexible, and reversible migratory traits (sensu Senner et al. 2015b). Complementary to the focus on individual behaviour, we encourage more research into the cumulative impact of weather conditions during migration on individual fitness. This will be greatly facilitated by studying migratory behaviour within the context of the entire annual cycle (Marra et al. 2015), combining the study of movement, eco-physiology and reproduction within a single system. Integrating empirical and theoretical approaches will empower us with tools to explore population level consequences of individual behaviour as well as eco-evolutionary dynamics in migratory species (Schoener 2011; Bauer and Klaassen 2013). However, studying the consequences of cumulative effects on survival for the numerous small migratory species (<50 g) is still a future challenge, because up to now, all the logging tags available for these small birds only report the story of survivors.

In the near future, products derived from operational weather radar networks may be available that quantify migratory activity at the continental scale in near real-time. While citizen science networks can help determine species composition of migratory fluxes observed by radar. Interdisciplinary research networks such as ENRAM (the European Network for the Radar Surveillance of Animal Movement) and BirdCast are working on moving these developments forward (Shamoun-Baranes et al. 2014; La Sorte et al. 2015a; Farnsworth et al. 2016). Combining knowledge about individual based behaviour with the quantification of spatial and temporal flows of migration will help answer macro-ecological questions regarding species distribution patterns (Kelly and Horton 2016). It will allow to quantify and perhaps predict the impact of migrants on ecosystem services and functioning (Bauer and Hoye 2014) and explore consequences of changing weather regimes in the future (La Sorte and Fink 2017). Radar-based detection tools and predictive models of migration based on local weather conditions already find important applications in the field of aviation safety to mitigate the consequences of bird-aircraft collisions (van Belle et al. 2007; Shamoun-Baranes et al. 2008) and insect pest detection and prediction (Leskinen et al. 2009; Drake and Reynolds 2012). Such tools could be improved by integrating information on behaviour derived from biologging data and designed for additional conservation, health and safety purposes, such as reducing the impact of wind farms on migration (Fox et al. 2006) and identifying priority areas for conservation (Buler et al. 2012), including the biosphere and the aerosphere (Diehl 2013; Davy et al. 2017). The major future challenge will be to bring together the huge amount of increasing information coming from numerous disciplines and researchers to achieve new fundamental insights into how internal and external factors shape the development of flexible migratory behaviour at individual level, patterns of migratory connectivity and life-history traits at population level, and the emergence of migratory fluxes and biogeographical distributions at a systems level.
